# A New Hope for Melasma: Long‐Term Clearance With 755‐nm Picosecond Alexandrite Laser and Topical JAK Inhibition

**DOI:** 10.1111/jocd.70495

**Published:** 2025-10-06

**Authors:** Ali Al‐Mamoori, Sajjad Ghanim Al‐Badri, Ibrahim Khalil, Wael Al‐Daraji

**Affiliations:** ^1^ Department of Dermatology Baghdad Teaching Hospital, Baghdad Medical City Baghdad Iraq; ^2^ College of Medicine, University of Warith Al‐Anbiyaa Karbala Iraq; ^3^ Dhaka Medical College and Hospital Dhaka Bangladesh; ^4^ Dermatology Department Ain Shams University Hospital Cairo Egypt

To the editor,

Melasma is a chronic, relapsing hyperpigmentary disorder with multifactorial pathogenesis, including melanocyte hyperactivity, inflammatory cytokine signaling, and photoinduction [1]. In Fitzpatrick skin types III–V, visible light (VL)–driven melanogenesis and post‐inflammatory hyperpigmentation make long‐term clearance difficult [2]. Conventional therapies such as hydroquinone, peels, and oral tranexamic acid often provide only transient improvement, with high relapse rates and PIH risk in darker phototypes. Novel strategies are needed.

We report a Fitzpatrick type IV woman, aged 31, with a 4‐year history of symmetrical malar‐type melasma refractory to hydroquinone, oral tranexamic acid, and superficial chemical peels. Examination revealed dense, patchy hyperpigmentation over the malar and zygomatic regions. Wood's lamp demonstrated a mixed epidermal–dermal pattern.

## 1 Treatment Protocol

A single session of 755‐nm picosecond alexandrite laser (PicoSure Pro; Cynosure, Westford, MA, USA) was performed (flat 10 mm, 0.25 J/cm^2^, 5 Hz; focus 6 mm, 0.7 J/cm^2^, 2.5 Hz, multiple passes) [3]. Post‐procedure care included fusidic acid + betamethasone cream (Fucicort, LEO Pharma, Ballerup, Denmark) twice daily for 3 days, followed by topical tofacitinib 2% cream twice daily for maintenance. She also applied an iron oxide–tinted sunscreen (Heliocare 360° Color Gel Oil‐Free; Cantabria Labs, Madrid, Spain) twice daily to protect against VL [2, 4] No bleaching agents were used.

## 2 Outcome

**FIGURE 1:**
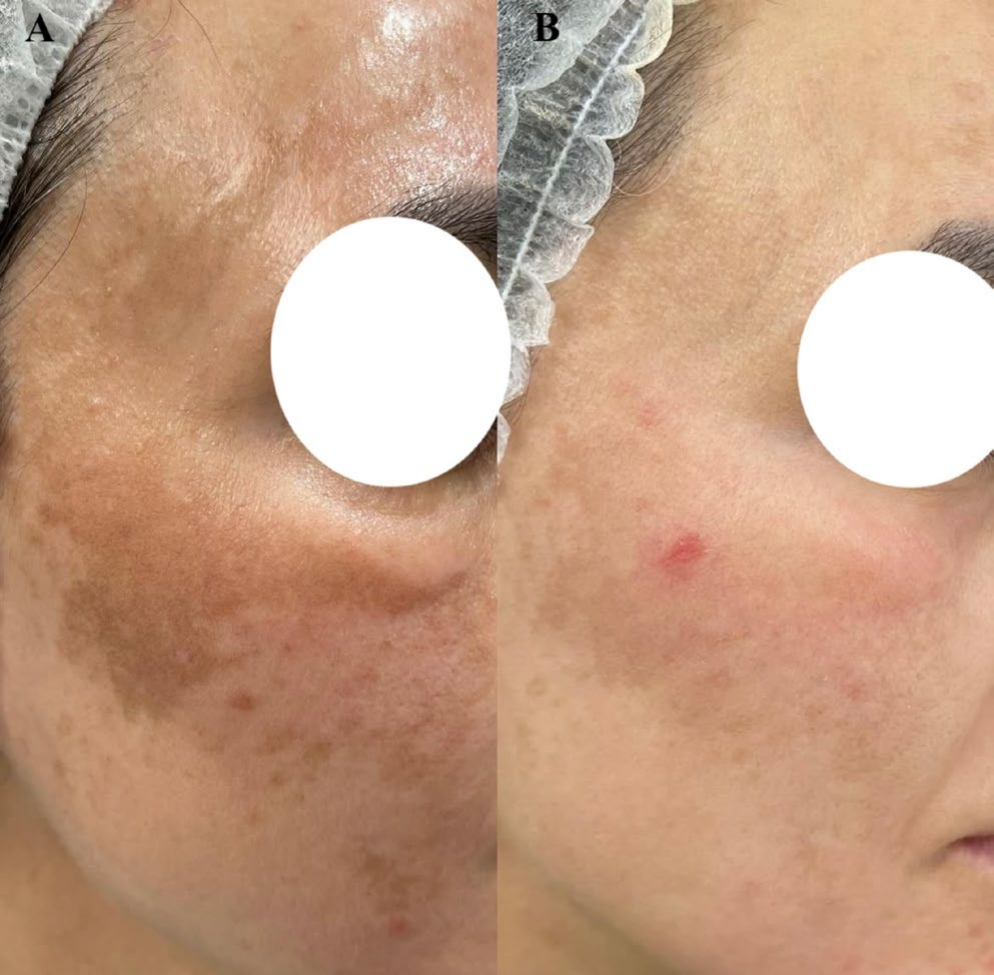
(A) Baseline: Dense, melasma affecting the malar and zygomatic areas. (B) Nine‐month follow‐up: Marked clearance after a single 755‐nm picosecond alexandrite laser session, maintained with topical tofacitinib and tinted sunscreen before gradual relapse.

Marked pigment clearance and homogeneous skin tone were observed (Figure 1A,B). Results were maintained for 9 months before gradual relapse. No adverse effects, post‐inflammatory hyperpigmentation, or rebound were reported.

## 3 Discussion

This case highlights the synergy of pigment disruption, inflammation control, and VL photoprotection:

- Pigment fragmentation—Picosecond pulses deliver photoacoustic melanin disruption with minimal thermal injury, reducing PIH risk [3].
- Inflammation modulation—Tofacitinib inhibits JAK1/3, downregulating IFN‐γ–driven keratinocyte–melanocyte cross‐talk implicated in melanogenesis and other pigmentary disorders [5].
- VL shielding—Iron oxide–tinted sunscreens block VL wavelengths that activate opsin‐3 and oxidative stress pathways, reducing recurrence risk [2, 4].

This strategy directly targets pigment overload, inflammatory signaling, and light‐induced oxidative stress simultaneously, offering a more comprehensive pathway to disease control. Importantly, clearance was achieved without hydroquinone or other bleaching agents, underscoring its potential relevance for darker skin phototypes where irritation and rebound are common.

## 4 Conclusion

This case provides early evidence that combining a 755‐nm picosecond alexandrite laser with topical JAK inhibition and visible‐light protection may offer a safe and durable approach for refractory melasma in darker phototypes. These findings warrant further investigation in larger controlled studies to validate efficacy and safety.

## Ethics Statement

Ethical approval was not required for this case report, as it describes the clinical course of a single patient without experimental intervention.

## Consent

Written informed consent was obtained from the patient's legal guardians for the publication of this case report, including clinical details and imaging findings, while ensuring confidentiality and anonymity.

## Conflicts of Interest

The authors declare no conflicts of interest.

## Data Availability

All data generated or analyzed during this study are included in this published article. No additional datasets are available.
